# Sperm Nuclear Vacuoles in relation to Acrosome Reactions and Sperm Motility

**DOI:** 10.1155/2014/178970

**Published:** 2014-07-03

**Authors:** Akira Komiya, Yoko Kawauchi, Tomonori Kato, Akihiko Watanabe, Ichiro Tanii, Hideki Fuse

**Affiliations:** ^1^Department of Urology, Graduate School of Medicine and Pharmaceutical Sciences for Research, University of Toyama, 2630 Sugitani, Toyama-shi, Toyama 930-0194, Japan; ^2^Department of Medical Biology, Graduate School of Medicine and Pharmaceutical Sciences for Research, University of Toyama, 2630 Sugitani, Toyama-shi, Toyama 930-0194, Japan

## Abstract

We investigated sperm nuclear vacuolation in relation to acrosome reactions and the maintenance of sperm motility. Thirty male patients who visited our Male Infertility Clinic were enrolled. These patients underwent conventional semen analyses, Acrobeads tests, and high-magnification observation of the sperm head to evaluate the degree of nuclear vacuolation on the Acrobeads test scoring after 24 hours of incubation. The presence of acrosome reactions was evaluated using the Acrobeads test. The spermatozoa were classified into three groups: (I) those bound to MH61-beads, (II) motile spermatozoa that did not bind to MH61-beads, and (III) immotile spermatozoa that did not bind to MH61-beads. The percentage of spermatozoa with large nuclear vacuoles (%LNV) was compared between the three groups. The degree of sperm nuclear vacuolation was evaluated in 17,992 ejaculated spermatozoa. The mean %LNVs were 2.4% in group I, 5.8% in group II, and 9.8% in group III. These values were significantly different from each other (*P* < 0.001, paired *t*-test). There were no correlations between the %LNV values and the Acrobeads scores. In conclusion, the degree of sperm nuclear vacuolation was significantly lower in the acrosome-reacted spermatozoa and spermatozoa with maintained motility, and higher in the immotile spermatozoa that did not bind to MH61-beads.

## 1. Introduction

In the field of assisted reproductive technology (ART), particularly that involving intracytoplasmic sperm injection (ICSI), one should select the single best motile spermatozoon, although the ability to identify fertile spermatozoa under a light microscope without denaturation is limited [1–4]. In this context, Bartoov's group reported that the ultramorphological status of subcellular organelles in the head is significantly associated with the outcomes of natural and IVF fertilization [5, 6]. This group further developed a method to assess the detailed morphology of motile spermatozoa in real time at a high magnification using a light microscope: the motile sperm organelle morphology examination (MSOME), used in the field of ART [7]. The examination is performed in real time using an inverted light microscope equipped with high-power Nomarski differential interference contrast (DIC) optics enhanced by digital imaging to achieve a magnification up to 6300×. MSOME has yielded a more strict definition of normal spermatozoa than conventional semen analyses, and new abnormalities, particularly sperm head vacuoles, have been described [8]. The high-magnification observation technique has been adapted to select the best spermatozoa for oocyte injection, introducing a new technique named intracytoplasmic morphologically selected sperm injection (IMSI). Morphological normalcy of the sperm nucleus, as defined by MSOME, is significantly and positively associated with both the rate of fertilization and the pregnancy outcomes [8]. In addition, the rate of pregnancy after IMSI is significantly higher than that observed following routine ICSI procedures (66.0% versus 30.0%) [9]. More recently, the results of ICSI with sperm selected according to conventional light microcopy or MSOME have been controversial, and the technique is thought to be time-consuming. However, a recent meta-analysis showed a trend towards an improvement in IMSI outcomes versus that obtained with ICSI [10]. To date, teratozoospermia has been proposed to be a preferential indication for MSOME and IMSI [11], while the only confirmed indication for IMSI is recurrent implantation failure following ICSI [12].

Numerous vacuoles have been identified inside the sperm nucleus under a light microscope at high magnifications. It is generally accepted that vacuolated spermatozoa are classified as having an abnormal morphology [13, 14]. Among MSOME parameters, the presence of a sperm nuclear vacuole is one of the most important findings. Recent results have shown that the presence of sperm nuclear vacuoles is negatively correlated with the rates of fertilization, pregnancy, and implantation [7, 8, 15–18]. Sperm vacuolation has also been reported to be negatively related to the parameters in conventional and computer-assisted semen analyses (CASA) [14, 19]. These findings suggest that the observation of sperm vacuolation can be used to predict the sperm function.

The status of nuclear vacuoles related to acrosome reactions has been investigated as a parameter of sperm function. To date, the results of investigations regarding the origins of vacuoles have been controversial, and, while some authors report vacuoles to originate within the nucleus, others report an acrosomal origin. It has been suggested that at least some nuclear vacuoles are of acrosomal origin [20, 21]. In addition, sperm head vacuoles are thought to be produced at earlier stages of sperm maturation and that normal acrosome reactions are more likely to be induced in spermatozoa without large nuclear vacuoles [22]. In this context, the Acrobeads test can be used to evaluate acrosome reactions in sperm using a monoclonal antibody that binds to the anterior portion of acrosome-reacted sperm [23, 24].

The purpose of the present study was to further investigate the relationship between sperm nuclear vacuolation and the sperm functions, including acrosome reactions, by conducting Acrobeads tests and assessments of the sperm motility status after 24 hours of incubation by a high-magnification microscope.

## 2. Materials and Methods

The Institutional Review Board of the University of Toyama approved this study (number 23-128). Ethical consent for the work to be carried out was provided, and signed informed consent was obtained from each patient evaluated in this study. The study conformed to the principles outlined in the Declaration of Helsinki.

### 2.1. Sample Collection

We enrolled 30 male patients who visited the Male Infertility Clinic at Toyama University Hospital. The patient ages ranged from 26 to 49 years with a mean of 36 years (±5.7, standard deviation). The duration of infertility ranged from 7 to 105 months with a mean of 37 months (±27, standard deviation). The semen samples were collected following masturbation from infertile male patients who visited the Male Infertility Clinic at Toyama University Hospital. The semen samples were collected after at least five days of abstinence, allowed to liquefy at room temperature, and evaluated within one hour of collection using manual conventional semen analyses [25], which were performed as previously described [26]. All manual assessments were performed by a single experienced laboratory technician (Y. K.), and the sperm concentrations were assessed using an improved Neubauer hemocytometer. The samples were diluted according to the instructions of the WHO laboratory manual (1999) [25]. To determine the degree of sperm motility, a 10 *μ*L sample was loaded onto a clear slide glass and covered with a 22 × 22 mm^2^ cover glass under a positive phase-contrast microscope at a total magnification of ×400. Male factors were generally screened based on medical history, physical examinations, conventional semen analyses, blood tests, including assessments of sex hormones and measurements of the testicular volume using an orchidometer, scrotal ultrasonography, and transrectal ultrasonography. Varicocele was diagnosed during scrotal examinations with the patient in a standing position and was graded as previously described [23]. Patients were excluded from this study if their final sperm concentration was <40×10^6^/mL after swim-up selection.

### 2.2. Acrobeads Test

Liquefied semen samples were diluted with an equal volume of modified Biggers, Whitten, and Whittingham (mBWW) medium at 37°C. The compound was centrifuged for five minutes at 200 ×*g*. The supernatant fluid was discarded, and 1.2 mL of mBWW medium was placed over the centrifuged sperm pellet. The centrifuge tube was kept at an angle of 5°  for one hour at 37°C, and motile sperm were collected from the medium using the swim-up method. One milliliter of the upper pole of the medium was removed and centrifuged for five minutes at 300 ×*g*. The pellets were washed twice in mBWW medium containing 0.3% human serum albumin (HSA) and then resuspended in mBWW medium containing 3.5% HSA at a sperm concentration of 40 × 10^6^ mL.

The Acrobeads test (FUSO Pharmaceutical Industries, Osaka, Japan) was performed according to the previously described method [23, 27] using immunobeads coated with MH61 monoclonal antibodies (MH61-beads) [28], which binds to the anterior portion of acrosome-reacted sperm. One hundred microliters of prepared sperm suspension was divided into a 96-well tissue culture plate (Corning, Corning, NY, USA). Serial dilutions were made with mBWW medium/3.5% HSA at 1 : 2, 1 : 4, and 1 : 8, and 10 *μ*L of mBWW medium/3.5% HSA including 1.5 × 10^4^ MH61-beads was added to each well. The plates were incubated at 37°C in 5% CO2 in humidified air. Agglutinated sperm-bead complexes were observed using an inverted phase-contrast microscope with 100x magnification at 24 hours of incubation. The visual field in each specimen was divided into five portions, and each field was considered to be positive when no beads free from binding to the sperm found were found. If three or more of the fields were positive, the well was judged to be positive. When less than three fields were positive in any well, the score was 0. Therefore, at least some spermatozoa were bound to MH61 even in the cases with a score of 0. When positive agglutination was observed at dilution of 1 : 1, 1 : 2, 1 : 4, or 1 : 8, the test results were scored as 1, 2, 3, or 4, respectively. To observe sperm vacuolation, the sperm suspension was placed onto a glass bottom dish (WillCo-Dish, WillCo Wells BV, Amsterdam, The Netherlands) instead of a 96-well tissue culture plate.

### 2.3. Observation of Spermatozoa Using a High-Magnification Microscope

**Figure 1:**
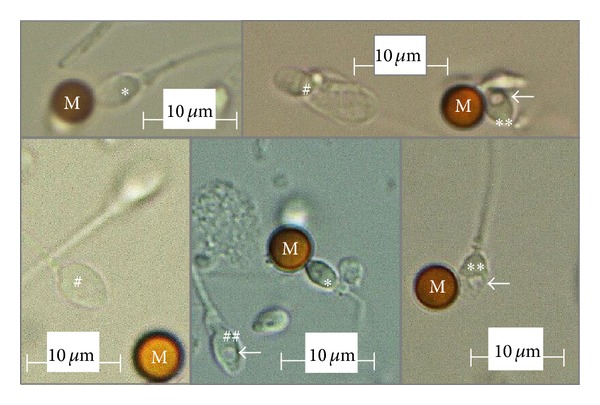
High-magnification observation of a sperm head (×600). M: MH61-bead; *spermatozoa bound to an MH61-bead without large nuclear vacuoles; **spermatozoa bound to an MH61-bead with large nuclear vacuoles; ^#^spermatozoa not bound to MH61-beads without large nuclear vacuoles; ^##^spermatozoa not bound to MH61-beads with large nuclear vacuoles. The arrows indicate sperm large nuclear vacuoles.

The spermatozoa placed on a glass bottom dish were analyzed at 3,700× using an inverted microscope equipped with Nomarski differential interference contrast optics (IX71, Olympus, Tokyo) and a video system (FX630, Olympus, Tokyo). A 60-× (1.42 numerical aperture) objective lens was used with oil. Images of the spermatozoa were captured and stored on a video system using an image-filing software program, FlvFs (Flovel, Tokyo). We spent 30 to 60 minutes capturing and analyzing the images of each ejaculate. The spermatozoa were classified into three groups: (I) those bound to MH61-beads after the acrosome reaction at 24 hours of incubation regardless of the motility, (II) motile spermatozoa that did not bind to MH61-beads, and (III) immotile spermatozoa that did not bind to MH61-beads. At least 500 spermatozoa per ejaculate and 100 spermatozoa per each group were evaluated using the high-magnification microscope [14]. A nuclear vacuole was defined as “large” if the maximum diameter of the vacuole was more than 50% of the width of the sperm head [14]. Using this system, we evaluated large nuclear vacuoles (LNVs) not only in motile spermatozoa but also in immotile spermatozoa (Figure 1).

The percentage of spermatozoa with LNVs was calculated for each sample and compared between groups I, II, and III.

### 2.4. Statistical Analysis

The statistical analysis of the data was carried out using the JMP 8.0.1 statistical software package (SAS Institute Japan, Tokyo). Paired and unpaired Student's *t*-tests were used to compare the values between the groups. The chi-square test was used to examine differences in categorical variables. Spearman's rank correlation coefficient was used to determine the correlations between the proportion of spermatozoa with large nuclear vacuoles (%LNVs) and the conventional semen parameters. A value of *P* < 0.05 was defined as being statistically significant.

## 3. Results

### 3.1. Conventional Semen Parameters and Acrobeads Test Results

The semen volume was 3.3 ± 1.7 mL (mean ± standard deviation), the sperm count was 53.5 ± 33.4 (×10^6^/mL), the proportion of sperm exhibiting motility was 43.7 ± 11.5%, and the proportion of sperm with a normal morphology was 5.0 ± 4.4% according to the conventional semen analysis. Conventional semen parameters were normal in 11 cases (including six patients with palpable varicocele) and abnormal in 19 cases (13 patients with palpable varicocele and six patients with idiopathic male infertility). The Acrobeads score was 0 in two cases, 1 in one case, 2 in 11 cases, 3 in 6 cases, and 4 in no cases. The scores in the patients with normozoospermia tended to be higher than those with teratozoospermia and/or asthenozoospermia; however, the difference was not significant (Table 1).

### 3.2. Observation of Spermatozoa Using a High-Magnification Microscope

High-magnification observation of spermatozoa was performed on a glass bottom plate at a dilution of 1 : 2 in 27 cases, a dilution of 1 : 4 in one case, and a dilution of 1 : 8 in two cases according to the final sperm count in the sperm suspension following the use of the swim-up methods and Acrobeads tests. The %LNVs (average ± standard deviation, minimum-maximum) were 2.4 ± 2.1% (0–8.1) in group I, 5.8 ± 3.9% (0.9–19.2) in group II, and 9.8 ± 4.3% (4.3–18.6) in group III. These values were significantly different from each other (I versus II, *P* < 0.001; I versus III, *P* < 0.001; II versus III, *P* < 0.001 paired *t*-test). There were no correlations between the %LNVs values and the Acrobeads scores (Table 2) or conventional semen parameters (data not shown) in this cohort.

## 4. Discussion

In the present study, we investigated the relationship between sperm nuclear vacuolation and the sperm functions, including acrosome reactions, by conducting Acrobeads tests and assessments of the sperm motility status after 24 hours of incubation by a high-magnification microscope. MSOME is a method used to evaluate motile spermatozoa; however, we applied a high-magnification microscope not only to motile spermatozoa but also to immotile ones. The %LNV varied according to the status of MH61-binding and motility after 24 hours of incubation at 37°C. The %LNV values in the spermatozoa that bound to MH61-beads after the acrosome reactions were significantly lower than those of the motile and immotile spermatozoa that did not bind to MH61-beads after 24 hours of incubation. Spermatozoa that bind to MH61-beads are thought to do so due to acrosome reactions. The %LNV values in the motile spermatozoa were significantly lower than those observed in the immotile spermatozoa after 24 hours of incubation. These results indicate that spermatozoa with LNVs are less likely to undergo acrosome reactions and maintain motility up to 24 hours at 37°C. The relationship between sperm nuclear vacuolation and sperm motility observed in the present study is consistent with the findings of our previous report [14]. In contrast, the %LNV values observed in this cohort were smaller than those noted in our previous report. This may be due to the differences in the patients' backgrounds, the semen quality, and/or methods used for sperm preparation. In the present study, patients with normozoospermia were included, and spermatozoa selection was performed using the swim-up method. On the other hand, the cohort evaluated in our previous study did not include normozoospermic patients, and the semen samples were processed using density gradient centrifugation. In this context, Monqaut et al. reported that the use of sperm processing methods, including swim-up method and density gradient centrifugation, allows for the selection of sperm with a lower level of nuclear vacuolization and a higher level of sperm motility [29]. In that study, the swim-up method produced samples with less vacuolization. In a report by Watanabe et al., the %LNV was 4.6% after both of the density gradient centrifugation and the swim-up method in high-quality semen samples, in which the mean values of sperm concentration and motility were 41.9 million/mL and 53.3%, respectively [30]. The definitions of LNV may also account for the %LNV. Our definition of vacuolated spermatozoa is different from and stricter than others [8, 13]. Therefore, the %LNV values observed in the present study are consistent with the findings of other reports.

As shown in Table 2, there were no differences in the %LNV values between the ejaculates with low and high Acrobeads scores. The spermatozoa bound to MH61-beads exhibited lower %LNV values, while those not bound to MH61-beads demonstrated higher %LNV values, regardless of the Acrobeads scores. The spermatozoa that lost motility at 24 hours of incubation also showed higher %LNV values, regardless of the Acrobeads scores. Spermatozoa with a normal function may be present in semen with abnormal Acrobeads scores. In contrast, the semen with abnormal Acrobeads scores may include the spermatozoa with both normal and abnormal functions. Based on these results, we speculate that the Acrobeads test reflects the quality of semen as a whole, whereas the %LNV reflects the degree of normality of the individual sperm functions. Therefore, it is sensible to select sperm according to the %LNV when performing ICSI.

Sperm vacuolation has been reported to be negatively related to parameters of conventional and computer-assisted semen analyses. The ratio of the vacuole area to the sperm head area is negatively correlated with a poor sperm morphology [19]. The proportion of large nuclear vacuoles in processed motile spermatozoa demonstrates significant correlations with decreased sperm count, sperm motility, total sperm count, motile sperm count, and total motile sperm count on conventional semen analyses [14]. In addition, the proportion of sperm with large nuclear vacuoles exhibits significant correlations with objective parameters of sperm motility, such as linearity and the beat/cross frequency measured using SMAS, a CASA system [14]. Varicocele repair reduces the proportion of large nuclear vacuoles in motile spermatozoa [31]. Therefore, the observation of sperm vacuolation can be used to predict the sperm function and evaluate the therapeutic effects.

The status of nuclear vacuoles related to acrosome reactions has been investigated as a parameter of the sperm function. Montjean et al. demonstrated that induced acrosome reactions are not correlated with significant modification of sperm nuclear condensation or morphology (Bartoov's criteria) [20]. The authors simultaneously observed a highly significant decrease in the presence of vacuoles following acrosome reaction induction. Kacem et al. evaluated the acrosome status using sperm organellar morphological examinations [21]. In that study, vacuoles were present in 61% of the spermatozoa when acrosomal material or intact acrosomes were observed, in comparison with the 29% observed when the spermatozoa were acrosome-reacted (*P* < 0.0001). In one study, the induction of the acrosomal reactions using ionophore A23587 from 17.4 to 36.1% significantly increased the percentage of vacuole-free spermatozoa from 41.2% to 63.8% (*P* < 0.001) [21]. These data suggest that some nuclear vacuoles are of acrosomal origin. Peer et al. investigated the effects of incubation at 37°C on the morphological normalcy of the sperm nucleus [32]. Their study showed that, after two hours of incubation at 37°C, there was a significant increase in the frequency of vacuolated nuclei. No significant morphological changes in sperm nuclei were observed after prolonged incubation at 21°C. Next, after two hours of incubation, the incidence of spermatozoa with vacuolated nuclei was significantly higher at 37°C than at 21°C. More recently, however, Neyer et al. reported that incubation temperatures (20 or 37°C) and/or the induction of oxidative stress do not stimulate the formation of new vacuoles [22]. In that study, after inducing the acrosome reactions, no modifications were detected in the vacuolated spermatozoa. These results suggest that some sperm head vacuoles are produced at earlier stages of sperm maturation and that normal acrosome reactions are more likely to be induced in spermatozoa without large nuclear vacuoles. In this context, the Acrobeads test can be used to evaluate acrosome reactions in the sperm using a monoclonal antibody that binds to the anterior portion of acrosome-reacted sperm [23, 24]. The results of tests using Acrobeads show good reproducibility and are correlated with the results of sperm penetration assays using zona-free hamster eggs and IVF [24, 33, 34]. Therefore, the acrosome status determined according to the Acrobeads test is a valuable parameter for estimating the capacity for fertilization in males with infertility. In our previous report, the Acrobeads score was found to be related to the sperm concentration and sperm motility in 81 ejaculates [27]. Komori et al. reported that sperm motility and the percentage of sperm with an abnormal morphology had an effect on the Acrobeads test results in 114 ejaculates [33]. In the present study, a similar trend was observed; however, it was not statistically significant. This may be due to the smaller sample size of 30 semen samples used in this study.

It is not clear how sperm vacuolation affects acrosome reactions and the maintenance of motility. The etiology of sperm nuclear vacuoles also remains unclear. Human sperm head vacuoles are physiological structures that are formed during the process of sperm development and maturation process [35]. Nuclear vacuoles may be the remnants of unnecessary cytoplasm and organelles that should have been eliminated during spermiogenesis [36, 37]. More recently, Perdrix et al. showed that vacuoles are located inside the nucleus using transmission electron microscopy [38]. Excess residual membrane constituents can be a source of reactive oxygen species (ROS). ROS expose sperm to excessive oxidative stress, resulting in DNA damage [39–44]. DNA damage is thought to reduce male fertility, and cause-specific treatments in patients with a high level of sperm DNA damage result in significant DNA improvement [45–53]. Many studies have indicated that there is a positive relationship between sperm DNA fragmentation and the presence of large nuclear vacuoles in the sperm nuclear area [53–55]. Furthermore, several studies have reported that large vacuoles are associated with chromatin condensation failure [38, 56–60]. DNA damage alters the special cellular functions of human spermatozoa, resulting in diminished acrosome reactions with reduced rates of fertilization. Ozmen et al. reported that negative correlations were identified between increased DNA damage and acrosome reactions and/or the viability of human spermatozoa, especially in cases involving reduced fertilization rates [61]. In addition, Morakinyo et al. reported that oxidative stress induced by calcium antagonists decreases the percentage value of acrosomal-reacted sperm in rats [62]. Therefore, the results in the present study can be explained by oxidative stress in spermatozoa and/or sperm DNA damage associated with LNVs.

There are some limitations to the present study. The number of ejaculates was relatively small. The results may have been different if we had obtained more samples, especially with respect to the relationships between the Acrobeads scores and semen quality or %LNV. However, our cohort was adequately large to analyze the differences in %LNV according to the presence of acrosome reactions and the maintenance of sperm motility. Acrobeads tests can be performed only in relatively high-quality semen processed using the swim-up method. Therefore, if the presence of acrosome reactions is evaluated using other methods with lower quality semen samples, the results will be different. No relationships in the sperm vacuolation or Acrobeads scores between pregnancy or birth rates were found, although only four of 30 couples achieved pregnancy, including two natural conceptions and two pregnancies via ICSI over a median follow-up of five months (data not shown). Such information would be beneficial in clinical practice. No lifestyle factors, including smoking, body mass index, and alcohol consumption, were found to be correlated with sperm vacuolation or the Acrobeads scores, although these factors may have had a potential negative impact on sperm vacuole development (data not shown). However, these factors have not been previously discussed in the literature.

## 5. Conclusions

In this study, the degree of sperm nuclear vacuolation was significantly lower in the acrosome-reacted spermatozoa and spermatozoa that maintained motility up to 24 hours of incubation and higher in the immotile spermatozoa that did not bind to MH61-beads. These results support the concept that the degree of sperm nuclear vacuolation evaluated using a high-magnification microscope reflects some of sperm functions.

## Figures and Tables

**Table 1:** Acrobeads scores and the results of the semen analysis.

Semen quality	Acrobeads scores		Chi-square test
	0-1	2–4	
Normozoospermia (*n*)	3	8	*P* = 0.1768
Teratozoospermia and/or asthenozoospermia (*n*)	10	9	

**Table 2:** Proportion of spermatozoa with large nuclear vacuoles according to the state of binding to MH61-beads and motility (*n* = 30).

	I. Spermatozoa bound to MH61-beads	II. Motile spermatozoa not bound to MH61-beads	III. Immotile spermatozoa not bound to MH61-beads
%LNV			
Mean ± SD	2.4 ± 2.1	5.8 ± 3.9	9.8 ± 4.3
*P* value (*t*-test)	<0.001∗ versus II, III	<0.001∗ versus I, III	<0.001∗ versus I, II
%LNV			
Acrobeads scores 0-1 (*n* = 13)	2.3 ± 0.6	5.5 ± 1.1	10.4 ± 1.2
Acrobeads scores 2–4 (*n* = 17)	2.5 ± 0.5	6.1 ± 1.0	9.4 ± 1.1
*P* value (*t*-test, 0-1 versus 2–4)	0.8088	0.6994	0.5506
Total number of observed spermatozoa	6474	5129	6389
Number of observed spermatozoa for each patient (mean ± SD)	215.8 ± 9.4	171.0 ± 44.3	213.0 ± 20.0

%LNV: proportion of spermatozoa with large nuclear vacuoles; SD: standard deviation. ∗Statistically significant.
